# Evaluation of periodontal condition and risk in patients with chronic kidney disease on hemodialysis

**DOI:** 10.1590/S1679-45082017AO3867

**Published:** 2017

**Authors:** Yeon Jung Kim, Luciana Martins de Moura, Christiane Peres Caldas, Caroline Perozini, Gilson Fernandes Ruivo, Debora Pallos

**Affiliations:** 1 Universidade de Santo Amaro, São Paulo, SP, Brazil.; 2 Universidade de Taubaté, Taubaté, SP, Brazil.

**Keywords:** Periodontal diseases, Risk, Renal insufficiency, chronic, Renal dialysis

## Abstract

**Objective:**

To establish a profile of periodontal conditions in chronic kidney disease patients on hemodialysis and their periodontal risk.

**Methods:**

We included 115 patients on hemodialysis. Clinical periodontal parameters assessed were: plaque index, gingival index, probing depth and clinical attachment level. Patients were classified according to presence/absence and severity of periodontal disease and periodontal risk.

**Results:**

In 107 dentate patients (93%) the plaque index was 1.53±0.78, the gingival index was 0.95±0.85, the probing depth was 2.2±0.6mm and the clinical attachment level was 3.18±1.75mm. We observed that 1 patient (0.94%) did not have periodontal disease, 55 patients (51.40%) had slight, 28 (26.17%) moderate and 23 (21.49%) severe periodontal disease. Among 107 patients, 37 (34.58%) had low risk, 35 (32.71%) moderate risk and 35 (32.71%) high risk. Patients with severe periodontal disease showed 104.5 more chance of high risk compared with low risk individuals (odds ratio: 104.5; 95%CI: 10.7-1017.2; p<0.0001).

**Conclusion:**

Most of patients with chronic renal disease presented periodontal disease, indicating the presence of chronic inflammatory and infection process that may influence in systemic conditions. A prevention and interventionist approach in this population is needed, especially to emphasize the importance of oral health. The periodontal risk assessment is a useful tool to create individualized periodontal therapies and to improve general health condition.

## RESUMO

**Objetivo:**

Traçar um perfil das condições periodontais de pacientes com doença renal crônica em hemodiálise e seu risco periodontal.

**Métodos:**

Foram incluídos no estudo 115 pacientes em hemodiálise. Os parâmetros clínicos periodontais avaliados foram: índice de placa, índice gengival, profundidade de sondagem e perda de nível de inserção clínico. Os pacientes foram classificados de acordo com a presença e a gravidade da doença periodontal, bem como de acordo com o risco periodontal.

**Resultados:**

Quanto aos parâmetros clínicos, 107 pacientes dentados (93%) apresentaram média de índice de placa de 1,53±0,78 e gengival de 0,95±0,85, profundidade de sondagem de 2,2±0,6mm e nível de inserção clínica de 3,18±1,75mm. Em relação à doença periodontal, 1 (0,94%) era saudável, 55 (51,40%) apresentavam periodontite leve, 28 (26,17%) moderada e 23 (21,49%) avançada. Com relação ao risco, dos 107 pacientes, 37 apresentavam risco baixo, 35 moderado e 35 alto. Os pacientes com doença periodontal avançada apresentaram 104,5 vezes mais chance de ter alto risco comparado ao baixo (*odds ratio*: 104,5; IC95%: 10,7-1017,2; p<0,0001).

**Conclusão:**

A maioria dos pacientes com doença renal crônica apresentou doença periodontal, indicando um processo infeccioso e inflamatório crônico, que pode influenciar na condição sistêmica. Evidencia-se a necessidade de uma abordagem preventiva e intervencionista nesta população, enfatizando a importância da saúde bucal. A avaliação do risco periodontal seria uma ferramenta na elaboração de terapias periodontais individualizadas para uma melhor condição de saúde geral.

## INTRODUCTION

The chronic kidney disease (CKD) constitutes a renal structural change (glomerular, tubular and endocrine) that is commonly progressive and irreversible and implicates in reduction or limitation of kidney’s ability to filter, therefore causing uremia (elevated levels of urea) characterized by accumulation of substances in the blood, which must be filtrated and expelled by kidneys.^1,2^ Uremia provokes immunodeficiency due to the increase of toxic substances in the bloodstream, therefore, patients with this disorder have suppression of humoral and immune responses. Some oral manifestations of CKD can be seen, such as xerostomy, uremic stomatitis, radiographic changes in maxillary bones and formation of dental calculus.^3^

In Brazil, there is an estimation of 499 patients per million on dialysis treatment, according to the census issued by the Brazilian Society of Nephrology in 2013, and CKD is considered a public health problem. A number of causes are associated with renal failure. The most common causes are *diabetes mellitus,* blood hypertension, chronic glomerulonephritis, polycystic kidney disease, among others. Around 61% of patients whom underwent dialysis had diabetes and hypertension.^4^ Balbo et al.,^5^ considered hemodialysis therapy a treatment that the goal is to improve systemic symptoms caused by accumulation of toxic substances.

Periodontal disease is an infection and inflammatory disease of tooth-supporting tissues, alveolar bone and periodontal ligament. Its main clinical characteristic is loss of insertion, mainly followed-up by periodontal pockets, and changes in density and at level of subjacent alveolar bone.^6-8^

Several hematological and genetic disorders have been associated with development of periodontitis and to the disease progression.^9^ Studies on pathogenesis of periodontal disease have shown the presence of periodontopathogenic bacteria by bacterial components such as lipopolysaccharide and endotoxin that can trigger an immune inflammatory response characterized by release of inflammatory mediators that are main associated factors with destruction of periodontal tissue. Periodontal disease was evaluated such as one with potential risk factors to mortality of patients in hemodialysis.^10-13^ We observed gram-negative microorganism derived from periodontal infection such as *Porphyromonas gingivalis, Tannerella forsythia,* Actinomyces actinocetomicomitans and Prevotella intermedia in bloodstream and we concluded that periodontitis can contribute significantly in evolution of systemic diseases.

On the other hand, periodontal therapy can be an impacting coadjuvant treatment for individuals who need hemodialysis. Vilela et al.,^14^ investigated influence of periodontal treatment of seric levels of C-reactive protein and interleukin 6 in patients with CKD. After 3 months we observed periodontal treatment that resulted in reduction of these inflammatory markers, therefore indicating an association of chronic periodontal disease and CKD. A reduction of c-reactive protein indexes was seen among hemodialysis patients 6 months after periodontal intervention.^15^

Chronic renal patients needed to have their risk evaluated and receive the correct diagnosis of periodontal disease to perform an adequate plan and maintenance therapy. Assessment model of periodontal disease risk has the aim to evaluate susceptibility, progression and prognosis of a disease in each individual.

Current literature lacks emphasis on the issue of periodontal disease risk in hemodialysis patients. Our study seeks to evaluate risk and develop an adequate treatment plan.

## OBJECTIVE

To create a profile of periodontal conditions of patients with chronic renal disease in hemodialysis and its periodontal risk.

## METHODS

This sectional study was approved by the Ethical and Research Committees of *Universidade de Taubaté*, protocol 0405/07, and *Faculdade São Lucas*, protocol 123/07.

We selected 115 patients with CKD who were on hemodialysis treatment at a nephrology clinic at Taubaté (SP), and nephrology clinic at Rondônia. All participants of this study were informed about proposals of the study and signed the consent form.

Inclusion were: agreement with the objective of the study, sign of the consent form, and patients who underwent hemodialysis. We excluded those who did not agree with objective of the study, patients who refused to sign the consent form, pregnant and lactating women, HIV seropositive patients and individuals with hepatitis B (HBV) and C (HCV).

Initially an anamnesis was performed to evaluate patient’s medical history (diabetes, use of tobacco products, and systemic diseases) and odontology of each individual followed by periodontal clinical exam. Periodontal clinical exam were done using two examiners previously calibrated (pondering Kappa=0.74) and periodontal probe Williams type (Trinity^®^, Brazil).

Clinical parameters considered were: plate index (PI), gingival index (GI), probing depth (PD), loss of clinical attachment level (CAL). Probing depth and CAL were evaluated in mesiobuccal, vestibular, distal-vestibular, mesiolingual, lingual and distal-lingual sites of each tooth, excluding soft molars.

Next, individuals were categorized according to presence and severity of periodontal disease based in established clinical criteria by the American Academy of Periodontology in mild, moderate and advanced periodontitis.^16^

Individual periodontal risk was determined using the assessment tool of modified periodontal risk developed by Chandra.^17^ Clinical parameters and individual information such as bleeding on probing, sites with PD ≥5mm, number of absent teeth, ratio of clinical attachment loss and age in addition to the presence of diabetes, smoking habit and systemic factor. These data were included in a program created in Microsoft Excel. After, individuals were categorized into three risk classes (low, moderate or high), based on functional diagram generated by the program.

The analysis of differences between periodontal disease and risk used a multivariate analysis of absolute frequency and χ^2^ test. According to obtained results we applied the odds ratio (OR).

## RESULTS

We included 115 patients in the study. Of these, 71 (61.74%) were men aged 47.30±18.35 years, and 16 (13.91%) were smokers. Mean time of dialysis was 3.43 years. Eight (6.96%) patients were totally toothless and 107 were dentate individuals (Table 1).

**Table 1:** Characteristics of the population

Variables	Mean±SD
Mean age, years	47.30±18.35
Sex, (%)	
Men	71 (61.74)
Women	44 (38.26)
Hemodialysis time, years	3.43±3.28
Smoking habit, (%)	
Smoker	16 (13.91)
Non-smoker	99 (86.09)
Diabetes, (%)	28 (24.34)
Toothless, (%)	8 (6.96)
Dentate, (%)	107 (93.04)

SD: standard deviation.

In terms of periodontal clinical parameters 107 dentate patients had mean of 18.36±7.95 teeth, mean of PI and GI, respectively, of 1.53±0.78 and 0.95±0.85, PD of 2.2±0.6mm and CAL of 3.18±1.75mm (Table 2).

**Table 2:** Periodontal clinical parameteres

Clinical parameters	Mean±SD
Gengival index	0.95±0.85
Plate index	1.53±0.78
Probing depth, (mm)	2.20±0.70
Clinical attachment level, (mm)	3.18±1.75
Dental presence, teeth	18.36±7.95

SD: standard deviation.

The table 3 presents an assessment of periodontal risk and periodontal condition of patients. In relation to periodontal disease, more than 99% of evaluated patients had any form of periodontitis, an individual (0.94%) was healthy, 55 (51.40%) had mild periodontal disease, 28 (26.17%) moderate periodontal disease, and 23 (21.49%) with periodontal advanced disease. The mild periodontal disease was more frequent compared to other groups, however, we did not observe differences among moderate periodontal disease and advanced periodontal disease (χ^2^, p<0.05).

**Table 3:** Evaluation of periodontal risk and periodontal disease

Risk of periodontal disease	Low	Moderate	High	Total
No periodontal disease	1 (0.93)A	0	0	1 (0.93)
Slight periodontal disease	30 (28.30)a*B*	22 (20.56)a*A**	3 (2.79)bA**	55 (51.45)
Moderate periodontal disease	5 (4.64)aA	10 (9.30)abB	13 (12.09)b*B	28 (26.03)
Advanced periodontal disease	1 (0.93)aA	3 (2.79)aC*	19 (17.67)b*B*	23 (21.39)

Total	37 (34.58)	35 (32.71)	35 (32.71)	107 (100)

Different letters indicates significant statistically difference (lowercase letters for lines and capital letters for columns).

* χ^2^, p<0.0001; ** p<0.05.

Of 107 patients, 37 (34.58%) had lower risk, 35 (32.71%) moderate risk and 35 (32.71%) high risk. We did not observe statistical difference between groups (χ^2^, p>0.05) (Table 3). Patients with mild periodontal disease had 20.8 times more chance of low risk compared with high risk patients (OR: 20.8; confidence interval 95% - CI95%: 5.7-74.7; p<0.0001). Patients with advanced periodontal disease had 104.5 times more chance of high risk compared with those with low risk (OR: 104.5; CI95%: 10.7-1017.2; p<0.0001).

## DISCUSSION

More than 99% of patients who were evaluated in the study had some form of periodontal disease that showed the need of periodontal interventional and maintenance therapies. Our results corroborate to previous studies that showed more prevalence of periodontal disease in renal compromised patients.^7,9,18^

Bastos et al.,^18^ reported higher severity of periodontal disease in CKD patients on hemodialysis associated with higher amount of periodontopathogen microorganisms. A worsening in oral hygiene conditions were also described, including higher plaque accumulation/dental biofilm, dental calculus, and bleeding gums in population of hemodialysis patients. The precarious condition can be justified by negligence, once oral hygiene would not be of high priority, highlighting the need of consciousness of this group of patients.^10,13,19^

Hemodialysis time can influence patients’ prognosis. Some studies show that periodontal disease become worse with hemodialysis time. Differently, we did not observe in this study the correlation of time of dialysis with extension and/or severity and risk of periodontal disease (not presented data).^3,20^

Despite relationship between periodontal disease and CKD is controversial in the literature, studies with interventional character show that periodontal therapies promote reduction of inflammation and reduce seric levels of inflammatory mediators, therefore influencing systemic parameters of the patient.^14,15,21^Recently, groups of patients with CKD who underwent non-surgical periodontal treatment have had improvement in interleukin-6 levels, ferritin and creatinine, and also in nutritional markers.^21^ The correct diagnosis and periodontal treatment would be of high valuable in hemodialysis patients, considering that inflammation and infection seen in oral cavity can contribute as a systemic focus.

Still, considering the periodontal risk model, we observed high prevalence of moderate periodontal disease and high to severe periodontal risk. When data of patients in hemodialysis were compared with clinical periodontal data, risk analysis showed coherence regarding patients with slight periodontal disease, classified as low risk, and patients with advanced periodontal disease, therefore showing high risk. This tool can made available a maintenance plan according to each degree of risk of the patient, therefore facilitating the day-by-day of dental surgeon, both clinical and periodontic. For this reason, correct diagnosis and periodontal treatment would be helpful in hemodialysis patients.

A number of models and methods have been proposed to periodontal risk assessment.^17,22^ The periodontal risk assessment was presented by Lang et al.,^22^ and it is one of most accepted models in the literature. We used the modified model risk assessment by Chandra,^17^ which had as advantages the easiness of software manipulation by target-public, the use of parameters and easy obtaining retrospective information, and results interpretation easiness. These two models of retrospective evaluation were recently compared and no significant difference was found, therefore showing that both models evaluated are effective in risk assessment.^23^

The majority of periodontal risk assessment models currently used helps to determine prognosis of periodontal disease and frequency of consultations in supporting therapy.^24^ A prospective study of 3 years associated the periodontal risk with dental loss and recurrence of a periodontal disease, estimating progression and determining intervals/frequency of consultation in supporting therapies, in addition to optimize treatment.^25^

Patients with advanced periodontal disease had greater chance of high periodontal risk (OR:104.5), which indicated positive correlation between risk and severity of periodontal disease in this group of patients. Therefore, assessment risk is highly important tool for planning interventional therapies and periodontal maintenance. A more frequent supporting therapy would be more interesting to improve periodontal condition that can influence their systemic condition.

A relevant aspect is that presence of periodontal disease can collaborate to progression of CKD. This additional factor can collaborate to begin renal replacement therapy and, for those in dialysis, it can influence negatively the loss of residual renal function, a reason that stimulate the diagnosis and treatment of periodontal disease.^26,27^

## CONCLUSION

We observed elevated prevalence of periodontal disease among nephropathy young men on hemodialysis and who had short-term hemodialysis treatment. The mild form of the disease is the most common. We also observed moderate to high risk of periodontal disease development in patients with chronic renal disease.

Presence of infectious and chronic inflammatory process, such as periodontal disease, can influence negatively the chronic renal disease progression, which is a reason for the need of multidisciplinary preventive and interventionist approach in this population, therefore emphasizing the importance of oral health. Periodontal risk assessment seems to be a tool to perform individualized periodontal therapies for better health condition.
